# Granulomatosis with polyangiitis presenting as axial proptosis and corneal perforation-a case report

**DOI:** 10.1186/s12886-025-04411-2

**Published:** 2025-10-22

**Authors:** Sanjana Bhatia, Teena Mariet Mendonca, Prathyusha Manikuppam, Anjali Sahni

**Affiliations:** 1 Dept. of Ophthalmology, Kasturba Medical College, Mangalore Manipal Academy of Higher Education, Manipal, Karnataka 575001 India; 2 Department of Rheumatology, Kasturba Medical College, Mangalore Manipal Academy of Higher Education, Manipal, India; 3 Dept of Internal Medicine, Kasturba Medical College, Mangalore Manipal Academy of Higher education, Manipal, India

**Keywords:** Necrotising vasculitis, Wegener’s granulomatosis, Ocular complications, ANCA-associated vasculitis, Autoinflammation, Blindness

## Abstract

**Background:**

Granulomatosis with polyangiitis (GPA) is a rare, aggressive, and rapidly progressive multisystem disease that primarily affects the respiratory tract, kidneys, and vascular system. Although less common, GPA can affect the eye through corneal involvement, scleral inflammation, and orbital mass formation. This report highlights a case in which sudden axial proptosis and corneal perforation revealed underlying GPA.

**Case presentation:**

A 32-year-old female presented with a 20-day history of painful left eye proptosis, purulent discharge, photophobia, and progressive vision loss, which eventually led to no perception of light at presentation. She also reported recurrent episodes of congestion in both eyes over two years, intermittent episodes of haematuria, and chronic cough.

On general examination, she was conscious and oriented to time, place, and person. Her vital signs were stable; however, she had pallor and bilateral chest crepitations. Ophthalmic examination revealed 6/6 vision in the right eye; however, the patient denied perceiving light in the left eye. The left eye exhibited axial proptosis, conjunctival congestion, chemosis, and a small corneal perforation with iris prolapse and a flat anterior chamber. A B-scan ruled out endophthalmitis, suggesting a non-infectious aetiology. On further imaging, CT orbit revealed a lacrimal fossa pseudotumor causing proptosis. HRCT of the chest revealed multiple cavitating lung nodules in both lungs.

Systemic evaluation revealed normocytic normochromic anaemia, leucocytosis, thrombocytosis, elevated ESR (87 mm/hr), CRP (156 mg/L), and rheumatoid factor (184 IU/mL). Urinalysis revealed haematuria and proteinuria, suggesting renal involvement. ANCA ELISA was strongly positive for anti-PR3 (338 U/mL), confirming a diagnosis of granulomatosis with polyangiitis (GPA). She was treated with topical and intravenous antibiotics and tissue adhesive with bandage contact lens application for corneal perforation. Systemic immunosuppression was achieved with intravenous methylprednisolone and cyclophosphamide. She was discharged on oral steroids with scheduled follow-up visits in the ophthalmology and rheumatology departments.

**Conclusion:**

This case highlights the importance of recognizing ocular signs as potential indicators of systemic vasculitis. Thus, timely diagnosis can help avert life-threatening systemic complications, thereby minimizing both the morbidity and mortality of the disease.

## Background

Granulomatosis with polyangiitis (GPA), also known as Wegener’s granulomatosis, is a systemic necrotizing vasculitis that targets small- to medium-sized blood vessels with a predilection for the respiratory tract, kidneys, and vascular system. Although ocular involvement is uncommon in comparison with systemic manifestations, conditions such as peripheral ulcerative keratitis (PUK), scleritis, and orbital masses are important presentations that pose a significant risk of blindness. [1]

This case report describes a case of sudden-onset axial proptosis and corneal perforation, which led to the diagnosis of an underlying, potentially life-threatening systemic autoimmune disorder.

## Case presentation

A 32-year-old female presented to our outpatient department with complaints of proptosis of the left eye, which was sudden in onset, painful, and progressive for the past 20 days. Proptosis was accompanied by conjunctival congestion, excessive purulent discharge, and diminished vision in the affected eye over the past 20 days, which progressed to complete vision loss in the last week. The patient also reported foreign body sensation, photophobia, and tearing, all of which progressively worsened during this period.

On further evaluation, she had a history of recurrent episodes of bilateral congestion and pain over the past two years, which temporarily subsided with the use of topical medications (unknown) prescribed by a local practitioner. Additionally, she reported intermittent episodes of haematuria, generalized weakness, and frequent respiratory infections, including chronic cough and nasal congestion.

On general physical examination, the patient was conscious, alert, and well-oriented to time, place, and person. Her vital signs were stable, with a blood pressure of 100/70 mmHg, a pulse rate of 76 beats/min (regular in rhythm and volume), a respiratory rate of 16 breaths/min, and an oxygen saturation of 99% on room air. Pallor was present, with no icterus, cyanosis, clubbing, lymphadenopathy, or pedal edema. Skin and mucous membranes were healthy, with no rashes, petechiae, or ulcers noted.

Examination of the paranasal sinuses revealed no tenderness over the frontal, maxillary, or ethmoidal sinuses bilaterally, and there was no associated swelling, nasal discharge, or postnasal drip. Respiratory system examination demonstrated bilateral crepitations, more pronounced in the right infraclavicular and axillary regions, as well as in the left mammary and infraclavicular areas. Breath sounds were vesicular without added wheeze, and percussion was resonant throughout. Cardiovascular examination revealed normal first and second heart sounds, with no murmurs, rubs, or gallops. The abdomen was soft and non-tender, with no organomegaly, masses, or free fluid, and bowel sounds were normal. Neurological examination showed intact higher mental functions.

**Fig. 1:**
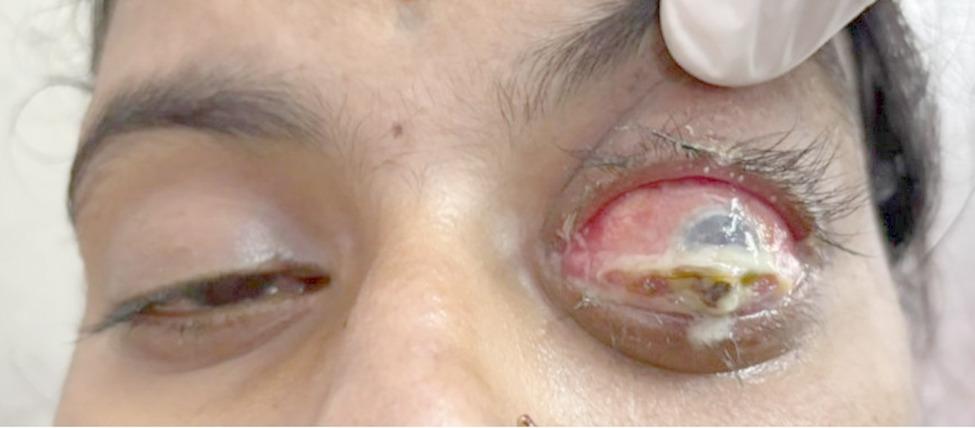
Clinical photograph of the patient showing axial proptosis and congestion of the left eye, along with a perforated corneal ulcer covered with purulent discharge

**Fig. 2:**
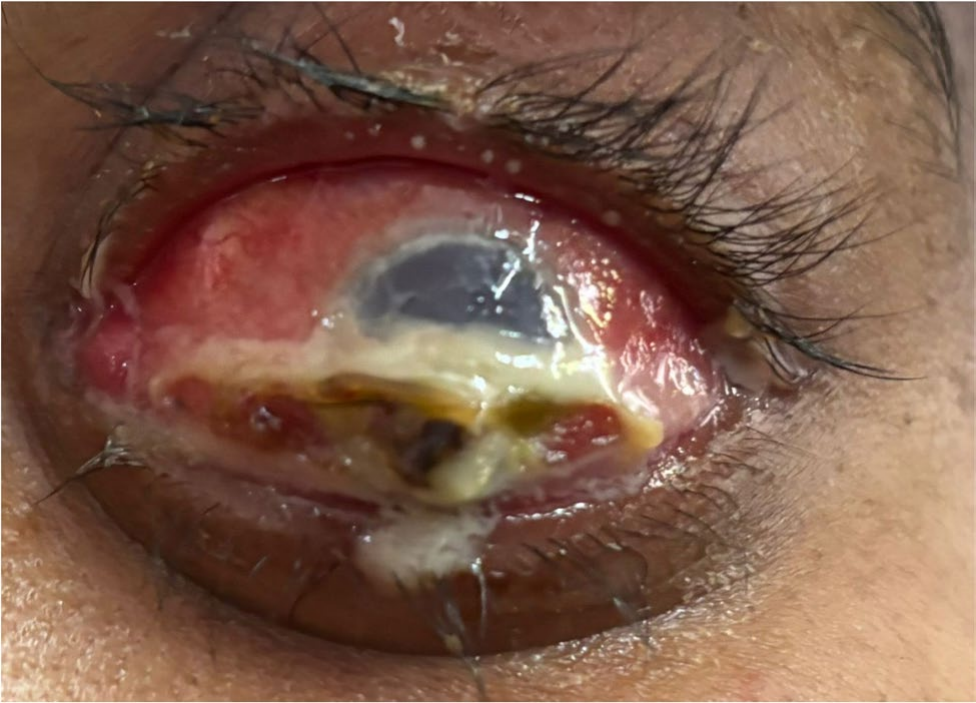
Close-up image of the left eye showing a perforated corneal ulcer covered with purulent discharge

On ophthalmic examination, the visual acuity of the right eye was 6/6, while the patient denied perceiving light in the affected left eye. Anterior segment examination of the left eye revealed severe axial proptosis, diffuse conjunctival congestion and pronounced chemosis (Fig. 1). A small full-thickness corneal perforation 3 mm in diameter extending from 12 to 3:00 was noted, with iris prolapse through the defect (Fig. 2). The anterior chamber was flat, suggesting significant aqueous leakage. Notably, there was no evidence of scleral thinning or overt scleritis.

Copious purulent discharge from the left eye initially raised a strong suspicion of an infectious aetiology such as endophthalmitis or pan-ophthalmitis secondary to orbital cellulitis. To evaluate the posterior segment, a B-scan ultrasonography was performed. The scan demonstrated an anechoic vitreous cavity with no dense vitreal echoes suggestive of vitritis, no evidence of choroidal thickening or choroidal detachment, and an intact, smoothly contoured retina without retinal detachment. The optic nerve head and posterior scleral contour appeared normal, with no signs of posterior scleral thickening or indications of orbital soft-tissue suppuration.

These imaging findings effectively ruled out features of intraocular extension such as endophthalmitis or pan-ophthalmitis and necessitated a re-evaluation of our initial working diagnosis of orbital cellulitis, directing clinical suspicion towards an underlying multisystem disorder as the principal driver of the ocular inflammatory process.

The right eye demonstrated an unremarkable anterior and posterior segment on examination, with no significant abnormalities.

Laboratory investigations revealed a haemoglobin level of 9.9 g/dL with a total leukocyte count of 12,950 cells/mm³ and an elevated platelet count of 7.04 lakhs/mm³, indicating leucocytosis and thrombocytosis. Peripheral smear examination revealed normocytic normochromic anaemia. The levels of inflammatory markers were markedly elevated, with an ESR of 87 mm/hr and a CRP level of 156 mg/L.

Urinalysis revealed trace proteinuria, haematuria (3+), and 13 pus cells per HPF. The urine protein-creatinine ratio was 0.62, suggesting renal involvement. Despite screening for an infective aetiology, including negative blood, urine, and sputum cultures, as well as a negative CBNAAT for tuberculosis, no infectious aetiology was identified. Rheumatoid factor was positive at 184 IU/mL, suggesting an underlying autoimmune process.

A definitive diagnosis was established with an enzyme-linked immunosorbent assay (ELISA), which revealed a markedly elevated anti-proteinase 3 (anti-PR3) ANCA level of 338 U/mL, confirming the diagnosis of granulomatosis with polyangiitis (GPA).

**Fig. 3:**
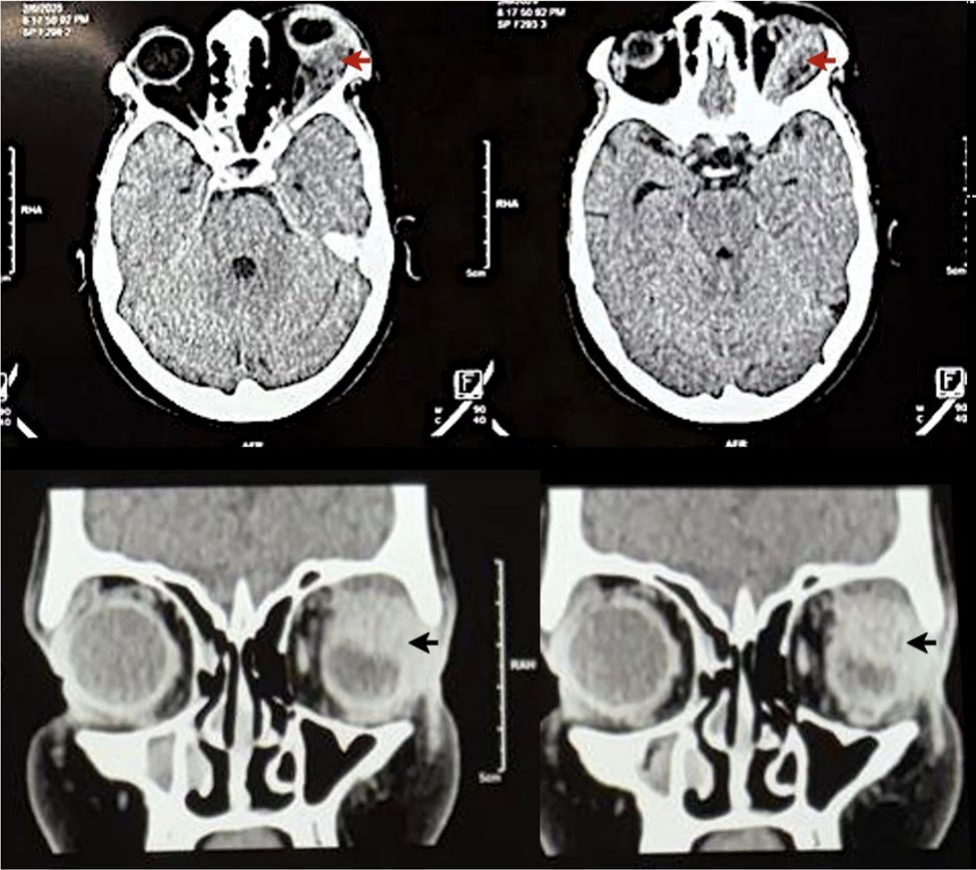
Contrast CT orbit images axial and coronal sections showing ill-defined trans spatial (involving extra and intraconal space) soft tissue density lesion (red and black arrows) measuring – 1.8 × 3.4 cm, epicentered in left lacrimal fossa showing no evident post contrast enhancement with adjacent conal fat stranding causing compression on the posterior segment of left globe and causing proptosis

Contrast-enhanced CT of the brain and orbit revealed an ill-defined soft tissue lesion (1.8 × 3.4 cm) in the left lacrimal fossa (Fig. 3). The mass caused axial proptosis and globe compression, with suspicious extension into the anterior temporal lobe convexity; these findings are consistent with orbital pseudotumor associated with GPA.

**Fig. 4:**
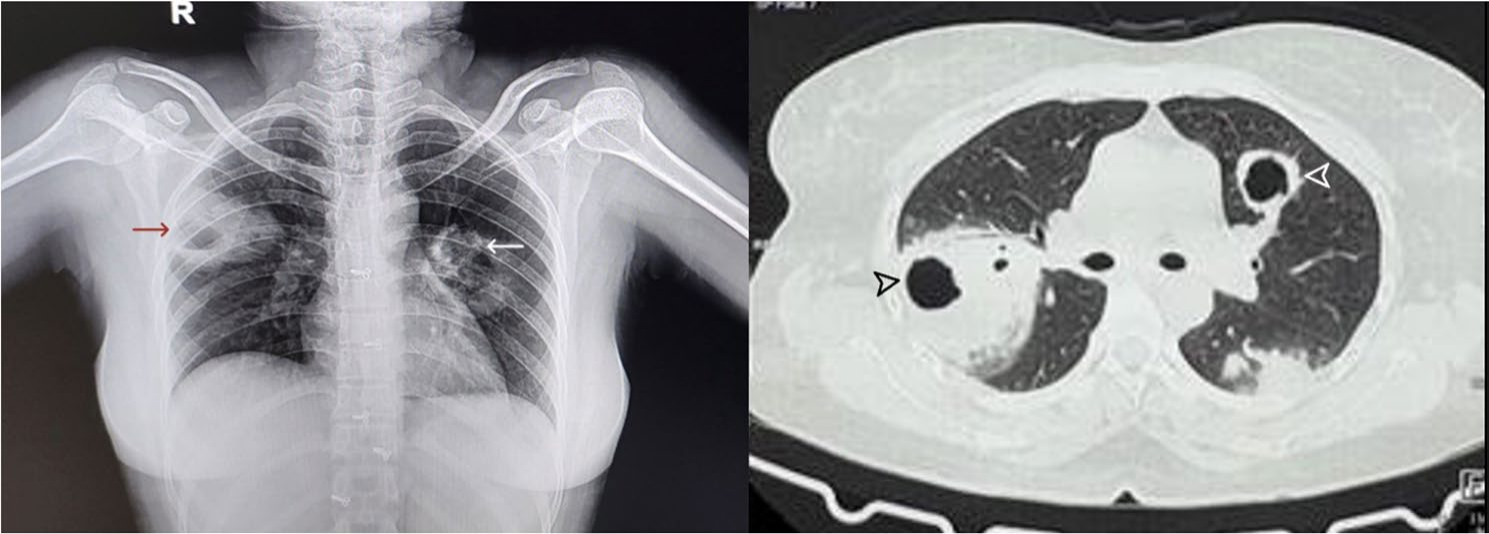
Chest x-ray and HRCT chest showing multiple large cavitating nodules in the posterior segment of the right upper lobe, superior lingular segment of left upper lobe, and superior segment of left lower lobe, with adjacent ground glass attenuation and soft tissue density nodules

HRCT revealed multiple large cavitating nodules with ground‒glass attenuation in both lungs (largest ~ 5 × 5.6 cm) and paratracheal lymphadenopathy (~ 14 × 9 mm), suggesting pulmonary involvement in GPA (Fig. 4). USG of the abdomen and pelvis revealed grade 1 renal parenchymal changes.

The patient was diagnosed with ANCA-associated vasculitis based on a strongly positive anti-PR3 ANCA panel. Ophthalmologic management included topical moxifloxacin antibiotic eyedrops administered six times daily in conjunction with systemic piperacillin–tazobactam (4.5 g, thrice daily for seven days). Corneal perforation was treated with tissue adhesive with bandage contact lens application.

**Fig. 5:**
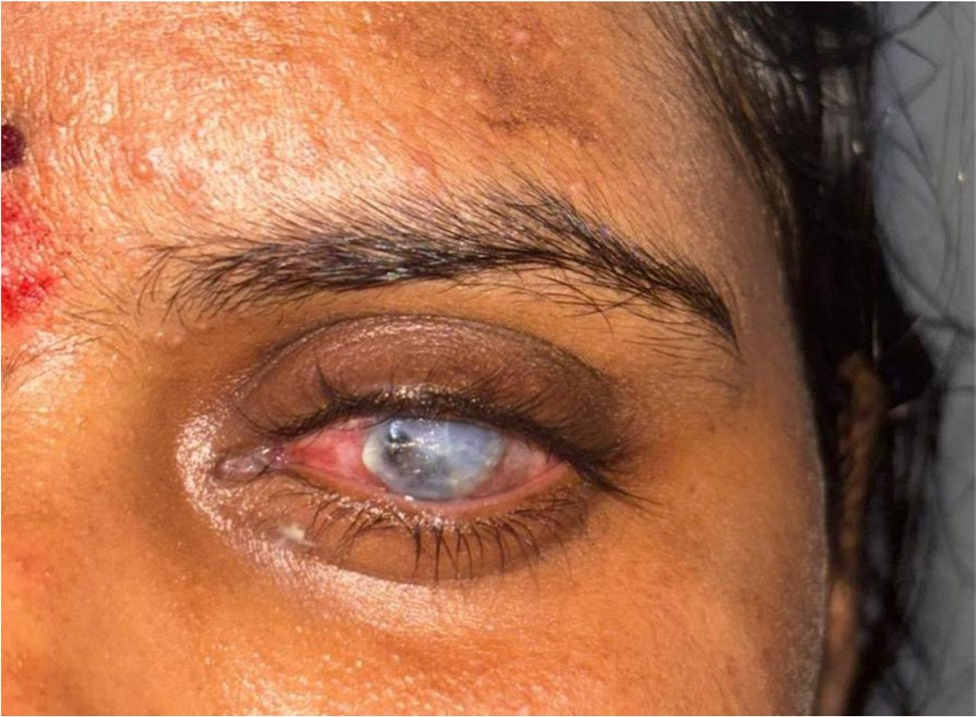
Clinical image of left eye showing resolved proptosis and healed corneal ulcer with pseudocornea

Rheumatological treatment involved methylprednisolone pulse therapy for 3 days, followed by dexamethasone (administered in a 4-4-2 mg regimen) and cyclophosphamide (15 mg/kg) as per the established protocol. Supportive care and intravenous antibiotics were continued during the hospital stay. The patient was discharged on 40 mg oral prednisolone once daily. At the one-month follow-up visit, her symptoms had improved drastically. Orbital inflammation and proptosis had resolved, and corneal inflammation had resolved with pseudocornea formation (Fig. 5).

## Discussion and conclusion

Granulomatosis with polyangiitis (GPA), previously known as Wegener’s granulomatosis, is a rare but potentially lethal vasculitis affecting multiple organs. Necrotizing granulomas that target small- to medium-sized vessels, damage them and surrounding tissues. It is classified as ANCA-related vasculitis alongside microscopic polyangiitis and Churg–Strauss syndrome. [2–4]

The disease typically affects individuals aged 45–65 years, although it can manifest at any stage, even in children [5]. This disease is mainly characterized by the presence of antineutrophil cytoplasmic antibodies (ANCAs), which are classified into two types based on how they appear under immunofluorescence: c-ANCAs (cytoplasmic) and p-ANCAs (perinuclear). Proteinase-3 (PR3), which is an enzyme found in neutrophils, is the main target site for c-ANCA, whereas myeloperoxidase (MPO), an enzyme stored in the granules of neutrophils and monocytes, is the target site for pANCA [6]. 

Ocular involvement in GPA is reported in 13–60% of cases, and in approximately 15% of cases, it may serve as the initial presenting symptom or can frequently emerge at various stages of disease progression. [7, 8]

Thus, timely diagnosis can help avert life-threatening systemic complications, thereby minimizing both the morbidity and mortality of the disease. [9]

As reported by Pakrou et al., scleritis, peripheral ulcerative keratitis (PUK), and orbital masses constitute the most common ocular manifestations, each of which carry a potential lethal risk of blindness [1]. 

In severe cases, the diagnosis is mainly clinical and requires a comprehensive medical, systemic and ocular examination. The 2022 ACR/European Alliance of Associations for Rheumatology (EULAR) classification criteria are the most widely accepted criteria for the diagnosis of GPA. The ELK classification system, introduced by DeRemee, categorizes GPA manifestations based on organ involvement: E for the ears, nose, and throat (upper respiratory tract), L for the lungs, and K for the kidneys, and a diagnosis is made in addition to a positive c-ANCA or a distinct histopathological finding indicative of GPA. [10]

Complete blood work, including ANCA testing, along with imaging techniques such as chest X-ray and high-resolution CT (HRCT), equally plays a crucial role in supporting the diagnosis.

Peripheral ulcerative keratitis (PUK), observed in our case report, occurs in 16.01% of GPA patients and represents a severe inflammatory disorder caused by immune-mediated destruction of the peripheral cornea, which, if untreated, can progress to perforation [11]. 

According to Hoffman et al., orbital involvement is the most common ocular manifestation of GPA, occurring in 45–50% of cases, followed by scleritis (16–40%) and corneal involvement (10–25%). [12]

While both rheumatoid factor and ANCA were positive in this patient, the overall clinical, radiological, and systemic profile was more consistent with ANCA-associated vasculitis, specifically granulomatosis with polyangiitis (GPA), rather than rheumatoid arthritis (RA)-associated systemic vasculitis. This conclusion was based on the following considerations:

The patient did not demonstrate clinical features typical of RA, such as symmetrical polyarthritis, joint swelling, or deformities, making RA as a primary diagnosis less likely. High-resolution computed tomography revealed multiple large cavitary pulmonary nodules, a radiological hallmark more frequently observed in GPA than in RA-associated lung disease, where such lesions are uncommon. In addition, the literature suggests that RA patients with ANCA positivity predominantly exhibit a usual interstitial pneumonia (UIP) pattern rather than cavitating nodular lesions, as supported by recent evidence [13]. The presence of proptosis with a soft tissue orbital mass is a recognized manifestation of GPA due to granulomatous inflammation. In contrast, orbital mass lesions are exceedingly rare in RA and, when present, are usually secondary to scleritis or episcleritis rather than true granulomatous masses. Taken together, these clinical, radiological, and systemic features strongly favored a diagnosis of GPA over RA-associated systemic vasculitis, despite the serological overlap.

The management of GPA typically begins with induction therapy, utilizing corticosteroids and immunomodulatory agents, followed by maintenance therapy with medications such as cyclophosphamide, glucocorticoids, rituximab, azathioprine, and methotrexate. In severe cases, plasmapheresis may be considered when indicated. [14]

Early intervention with immunosuppression is crucial in halting disease progression and preventing irreversible vision loss. In this patient, the presence of axial proptosis and corneal perforation alongside pulmonary and renal involvement highlighted the systemic nature of her disease.

## Data Availability

More details are available on request (contact details: Dr Teena Mendonca, Department of Ophthalmology, Kasturba Medical College Mangalore, Karnataka, India-575001. Email: tmendonca87@gmail.com).

## Abbreviations

GPA: Granulomatosis Polyangiitis
CT: Computed tomography
HRCT: High-resolution computed tomography
CRP: C-reactive protein
ESR: Erythrocyte sedimentation rate
ANCA: Antineutrophil cytoplasmic antibodies
c-ANCA: cytoplasmic antineutrophil cytoplasmic antibodies
p-ANCA: perinuclear antineutrophil cytoplasmic antibodies
PUK: Peripheral ulcerative keratitis
CBNAAT: Cartridge-based nucleic acid amplification test
ELISA: Enzyme-linked immunosorbent Assay
